# Between the first and second wave of the 2019 coronavirus pandemic (COVID-19): Presentation and crowding of attenders for mentale disorder and intossication/substance abuse

**DOI:** 10.1192/j.eurpsy.2021.976

**Published:** 2021-08-13

**Authors:** G. Savioli, S. Pesenti, I. Ceresa, E. Oddone, M.A. Bressan

**Affiliations:** 1 Emergency Department, IRCCS Policlinico San Matteo, Pavia, Italy; 2 In Cammino Social Cooperative Of San Pellegrino Terme (bg)., La Bonne Semence social cooperative of Oltre il Colle (BG), Bergamo, Italy; 3 Department Of Public Health, University of Pavia, Pavia, Italy

**Keywords:** COVID-19 pandemic, metal disorder, Emergency department, intossication and substance abuse

## Abstract

**Introduction:**

During the 1st wave of CoViD-19 pandemic there was a drastic reduction in total number of accesses, with more serious cases and a exorbitant increase in crowding, due to access block.

**Objectives:**

evaluate population who went to ED for (1) mental disorders requesting a psychiatric visit and for (2) intossication and substance abuse, between the first and second wave of the coronavirus pandemic

**Methods:**

We enrolled all patients who went at our ED from May 1 to October 20, 2020 and during the same period of 2019. We analized: vital parameters, age, sex, exit severity codes, hospitalization rate, Crowding input factors (number of access, waiting time, priority time to doc), Crowding throughput factors (LOS: Length Of ED Stay), Crowding output factors (percentage of access block; Total Access Block Time).

**Results:**

The results are shown in table 1

**Table:** 

Table 1	Mental-disorder		intossication/substance-abuse	
	May1-October 20,2020	May1- October 20,2019	May1-October 20,2020	May1- October 20, 2019
number of ED access	543	564	182	254
higher (yellow and red) priority time to doc (%)	28%	29%	50%	39%
worse exit severity codes (%)	10%	6%	16%	11%
rate of hospitalization (%)	26%	20%	16%	9%
average waiting times (min)	60	64	76	79
LOS lenght of stay (min)	369	326	629	506
access block (%)	3%	2%	5%	4%
Total Access Block Time: examination rooms (min)	11.538	8.384	8.059	8.889
Total Access Block Time: holding area (min)	8.382	3.963	182	254

**Conclusions:**

We would like to thank all employees of the IRCCS Policlinico San Matteo Foundation for their extraordinary efforts during the pandemic.

